# Supplementary figures and images for: Isolation, Characterization, and Autophagy Function of BECN1-Splicing Isoforms in Cancer Cells
Source: Biomolecules. 2022 Aug 2;12(8):1069. doi: 10.3390/biom12081069 (PMC9405542; doi:10.3390/biom12081069)

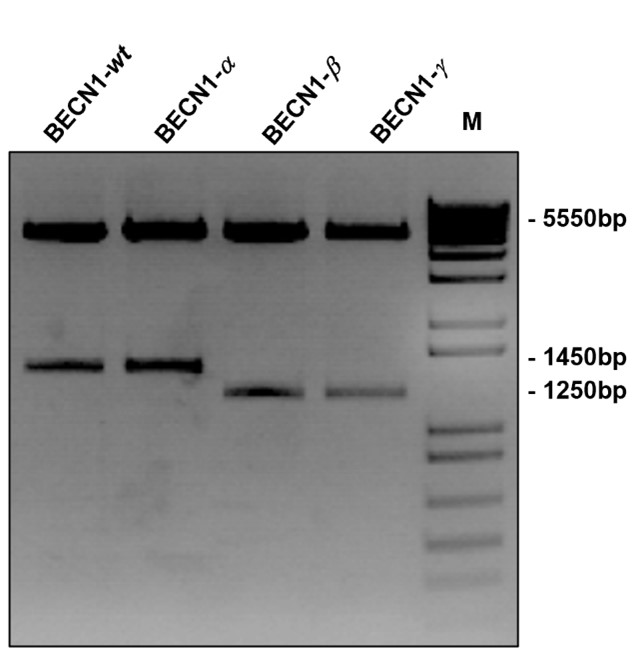

Supplement: Supplementary file 1 [file biomolecules-12-01069-s001.zip › biomolecules-1829267-supplementary_final proofed version/Figure S1.jpg]

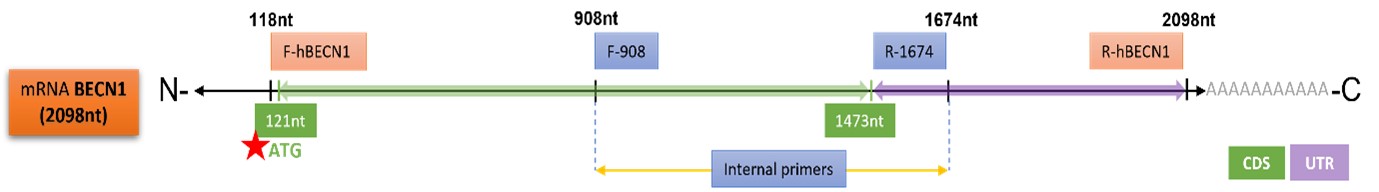

Supplement: Supplementary file 1 [file biomolecules-12-01069-s001.zip › biomolecules-1829267-supplementary_final proofed version/Figure S2.jpg]

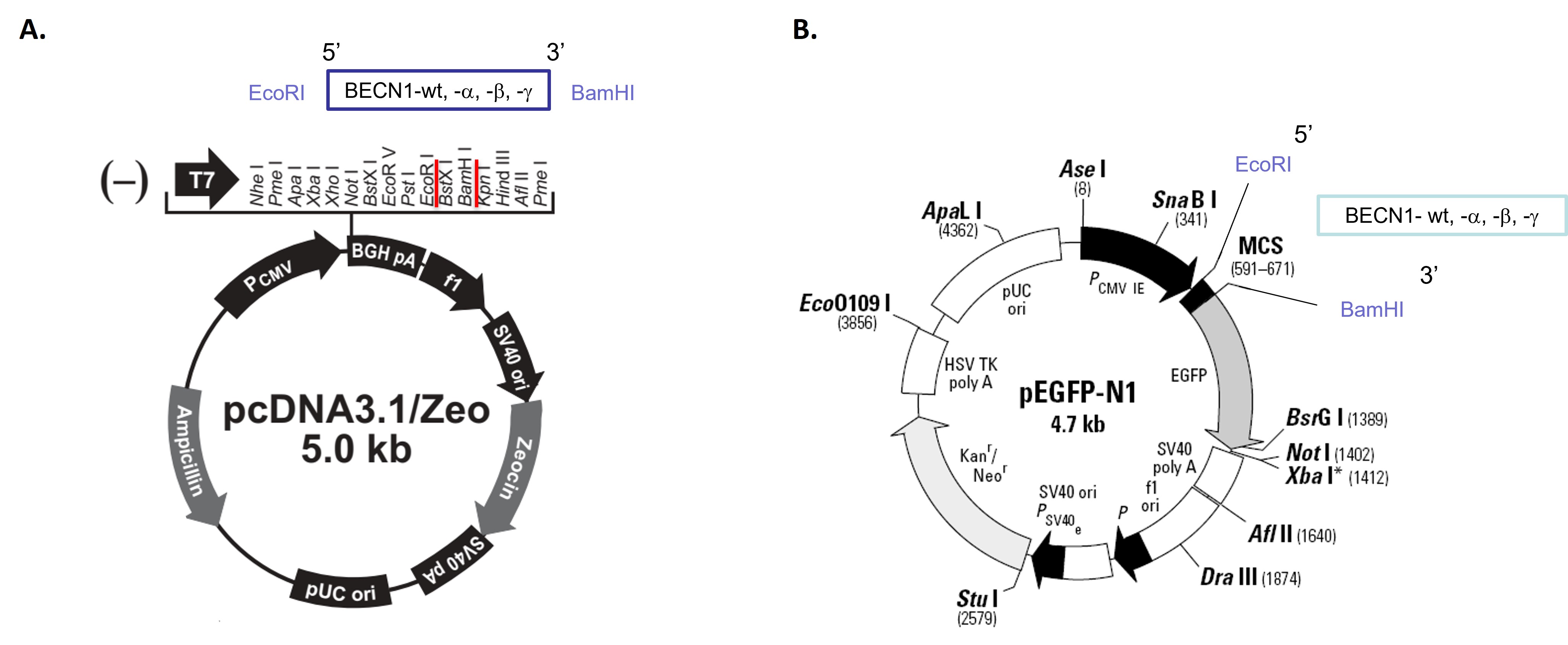

Supplement: Supplementary file 1 [file biomolecules-12-01069-s001.zip › biomolecules-1829267-supplementary_final proofed version/Figure S3.jpg]

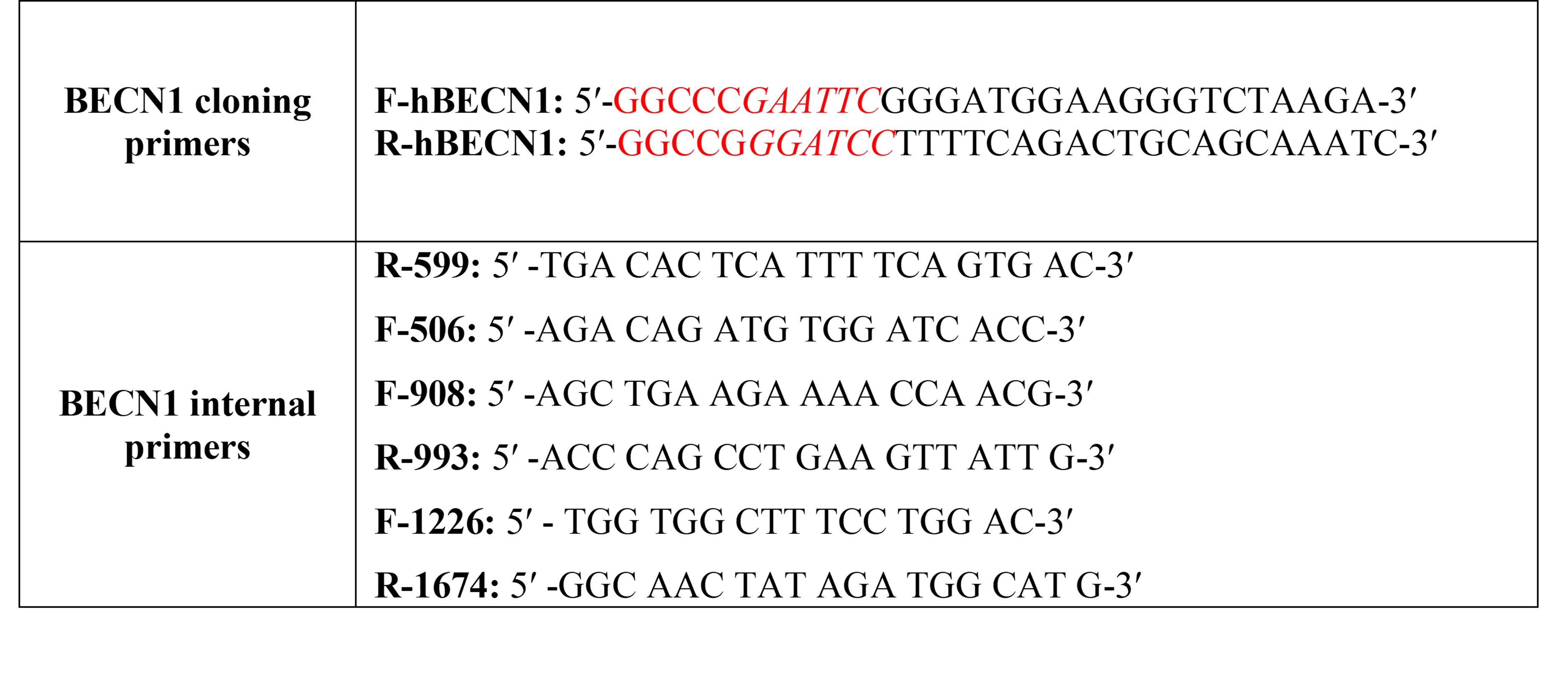

Supplement: Supplementary file 1 [file biomolecules-12-01069-s001.zip › biomolecules-1829267-supplementary_final proofed version/Table S1.jpg]
